# P-401. Stenotrophomonas maltophilia in Infants in a Cardiovascular Intensive Care Unit

**DOI:** 10.1093/ofid/ofaf695.618

**Published:** 2026-01-11

**Authors:** Chrisevelle Leigh Heller, Asma Razavi, Grant Stimes, Elizabeth Tocco, Lea Fasciano, Duc Nguyen, Lucila Marquez, Judith R Campbell

**Affiliations:** Baylor College of Medicine, Houston, TX; Baylor College of Medicine, Houston, TX; Texas Children's Hospital, Houston, TX; Texas Children's Hospital, Houston, TX; Texas Children's Hospital, Houston, TX; Baylor College of Medicine, Houston, TX; Baylor College of Medicine, Houston, TX; Baylor College of Medicine, Houston, TX

## Abstract

**Background:**

Infants in cardiac intensive care units (CICU) are at increased risk for healthcare-associated infections. *Stenotrophomonas maltophilia* (*S. maltophilia*) is an emerging pathogen in critically ill patients.

**Methods:**

We sought to describe patient characteristics and risk factors for acquiring *S. maltophilia* in infants hospitalized in our CICU. We performed a retrospective, case-control study of patients (≤ 1 year of age), in our CICU from 2020 –2023. Cases had a culture that grew *S. maltophilia* from any source and were matched to 3 controls based on age and sex. Demographic, clinical, microbiologic, and antibiotic data were abstracted from the electronic health record. Data were reported as frequencies and proportions for categorical variables and as mean and standard deviation for continuous variables. Characteristics and risk factors were analyzed using univariable conditional logistic regression model. All-cause mortality within one year from admission was estimated using the Kaplan-Meier statistic.

**Results:**

We identified 19 cases with *S. maltophilia* and 57 controls. There were no differences in demographic characteristics or congenital heart defect between cases and controls. Similar proportion had indwelling devices and respiratory support. Cases were significantly more likely to have a tracheostomy, OR 9.59 (95% CI 1.97, 46.62; p=0.01) during the hospitalization. Cases had a longer length of stay, OR 1.10 (95% CI 1.00, 1.10; p=0.047). Although total antibiotic days were similar, cases had three times more cefepime days, OR 1.06 (95% CI 1.01, 1.12; p=0.02) than controls. Crude all-cause mortality was higher in cases, OR 5.96 (95% CI 1.48, 24.03; p=0.01) and in infants with birthweight < 1500 grams, OR 3.99 (95% CI 1.22, 13.06; p=0.02).

**Conclusion:**

Presence of a tracheostomy and exposure to cefepime were associated with acquisition of *S. maltophilia* in infants in our CICU. Our findings highlight the importance of antimicrobial stewardship efforts targeting broad spectrum antibiotics, such as cefepime.

**Disclosures:**

All Authors: No reported disclosures

